# Retraction: An analysis of machine learning risk factors and risk parity portfolio optimization

**DOI:** 10.1371/journal.pone.0323221

**Published:** 2025-05-07

**Authors:** The PLOS One Editors

The *PLOS One* Editors retract this article [1] due to concerns about potential manipulation of the publication process. In addition, the article contains multiple uses of terminology that differ from standards in the field (e.g., “head-part analysis (PCA)” instead of “principal component analysis”).

These concerns call into question the validity and provenance of the reported results. We regret that the issues were not identified prior to the article’s publication.

LW, MA, and KR did not agree with the retraction. SAQ and YAK either did not respond directly or could not be reached.
